# Platelet-Rich Plasma and Fractional CO_2_ Laser Therapy to Reduce Surgical Intervention for Symptomatic Vaginal Mesh-Related Complications

**DOI:** 10.1007/s00192-025-06123-z

**Published:** 2025-04-03

**Authors:** Nadia Willison, Connor McPhail, Elvis Seman, Mahshid Taheri, Pouria Aryan, Tran Nguyen, Johnny Yi, Derek Abbott, Tanaka Dune, Fariba Behnia-Willison

**Affiliations:** 1 Discipline of Biomedical Engineering, School of EME, The University of Adelaide, Adelaide, South Australia Australia; 2 Flinders Medical Centre, Flinders University, Bedford Park, South Australia Australia; 3 FBW Gynaecology Plus, Ashford, South Australia Australia; 4 Department of Medical and Surgical Gynecology, Mayo Clinic, Phoenix, Arizona USA; 5 Faculty of Medicine, Dentistry and Health Sciences, University of Melbourne, Melbourne, Victoria Australia

**Keywords:** CO_2_ laser, Pelvic organ prolapse, Platelet-rich plasma, Vaginal mesh, Vaginal mesh complications, Vaginal mesh exposure

## Abstract

**Introduction and Hypothesis:**

Mesh implants were used in Australia until 2018 for managing pelvic organ prolapse. Owing to complications such as dyspareunia, mesh exposure, erosion and vaginal discharge, transvaginal mesh was removed from the market. Regenerative treatments such as transvaginal platelet-rich plasma (PRP) and fractional CO_2_ laser therapy may offer relief from mesh complications.

**Methods:**

From 2013 to 2024, women with mesh complications, including dyspareunia, vaginal discharge and/or vaginal mesh exposure (< 2 cm) awaiting surgery, were enrolled in this prospective study. Wilcoxon signed-rank test was used to assess IQR changes in Australian Pelvic Floor Questionnaire (APFQ) and Pelvic Organ Prolapse Quantification symptom severity, whereas a general linear model analysed outcome differences at baseline, 3–6 months and > 9 months. The primary aim was to assess the proportion of patients who avoided surgical intervention after PRP and CO_2_ laser treatment.

**Results:**

A cohort of 47 women were eligible. The average age and body mass index were 64 years and 27.94 kg/m^2^ respectively. Thirty-nine received PRP and CO_2_ laser combined, whereas 8 underwent CO_2_ laser alone. Overall, 40 women (85.0%) avoided surgery over an average 12-month follow-up. APFQ scores improved significantly from baseline to > 9 months (*p* = 0.02). Treatments also improved bladder, bowel, prolapse and sexual function (*p* < 0.001) between 3–9 months. Vaginal laxity and prolapse sensation improved at 9 months (*p* = 0.04, *p* = 0.005).

**Conclusions:**

Platelet-rich plasma and CO_2_ laser treatments allowed most women to avoid surgery, improving bladder, bowel, sexual function and vaginal atrophy. These alternatives may expand treatment options for mesh complications.

## Introduction

Pelvic organ prolapse (POP) is defined as a progressive pelvic organ herniation through the urogenital diaphragm, which affects up to 50% of parous women [1]. Ageing, an increased body mass index (BMI), vaginal multi-parity, assisted delivery, menopause, hysterectomy, smoking, chronic constipation and chronic respiratory disease are amongst the well-known risk factors for POP [1] . In an ageing population experiencing increasing levels of obesity, combined with an expectation for a better quality of life, the demand for surgical correction of POP is estimated to increase by 50% over the next 40 years [2]. Across history, a variety of surgical solutions for treating POP have been proposed, yet none more controversial than synthetic mesh.

Differing approaches to mesh implantation techniques along with different mesh kits have been studied for efficacy, safety and adverse events. However, over the past 15 years, a serious and necessary focus has centred on mesh-related complications affecting women worldwide [3]. The first collective large-scale reports of serious issues associated with mesh were first noted in those women who had undergone transvaginal mesh (TVM) procedures for the treatment of POP. Complications including mesh exposure and/or erosion, abnormal vaginal discharge, odour, vaginal bleeding, infection, voiding dysfunction, dyspareunia and significant pelvic pain occur most commonly in the first postoperative year but can manifest years after surgery [3]. Further, TVM kit recalls occurred worldwide where TVM was removed from the market by the Therapeutic Goods Association in 2017 [4] and the Food and Drug Administration in 2019 [5] owing to the effect of these complications on the lives of women. The TVM ban has affected the availability of other known safe and efficacious forms of mesh placement, including abdominal mesh for POP, and retropubic mesh for stress urinary incontinence (SUI) in some countries [6]. A clear understanding of both patient clinical characteristics and the material properties of pelvic mesh that improve its safety profile, thereby reducing complications related to exposure, is necessary.

Large pore size in pelvic-mesh design facilitates tissue integration and reduces infection risk by preventing pore deformation. Additionally, minimising microbial contamination during the placement of transvaginal urethral tapes is crucial for infection prevention [7]. Mesh incorporation into host tissue relies on the natural acute inflammatory reaction of the body, neovascularisation, cellular infiltration, tissue remodelling, then fibrosis, generally within 2 years of implantation [8].

Risk factors for mesh exposure include advancing age, menopause, the severity of the prolapse, oestrogen deficiency, smoking, obesity, implant size, graft-material properties, tissue compatibility and operative technique [9]. Urogenital atrophy, sexual activity and diabetes may increase the risk of mesh exposure [10]. Dyspareunia and a high risk of mesh exposure include previous damage to the vaginal vasculature (related to surgeries, scars, interrupted sutures, mesh size, or haematoma formation), excessive tension in sutures and decreased tissue perfusion [8, 10]. Pathophysiologically, exposure is due to an excessive inflammatory reaction or bacterial colonisation [10], causing inadequate or compromised epithelial healing [8]. The risk of mesh exposure is 4.2% after suburethral sling [10], 4.0–19.0% after TVM POP repair [11] and 3.5–10% after mesh sacrocolpopexy [12, 13]. Small mesh exposures without infection can be treated with vaginal oestrogen with or without local mesh excision depending on patient symptoms and sexual-activity status [3]. Despite its safety and efficacy, there exists an important cohort of women needing treatment who continually decline vaginal oestrogen owing to their perception of its ability to lead to breast cancer. A study by Unger et al. reported that more than half of patients who had a mesh complication underwent surgical management, a third of patients required conservative therapy after surgery and 8.0% required a second surgery [14]. Major complications such as severe pain, dyspareunia, recurrent exposures and/or viscous injuries often require complex extended or complete mesh removal, performed in tertiary centres by experienced surgeons in a multidisciplinary team setting [3, 14].

Although surgery is one of the mainstays of treatment, alternative methods of treating mesh exposures is necessary, with the goal of providing women with increased effective options for treating their tissue. Platelet-rich plasma (PRP) is defined as a plasma preparation containing a higher concentration of platelets than physiological levels [15]. PRP promotes tissue revitalisation and angiogenesis at the site of the injury, leading to remodelling and epithelialisation [15]. Early clinical studies show that PRP may have promise in the treatment of conditions such as lichen sclerosis [16] and urinary incontinence [17]. Additionally, PRP may assist in revitalising ovarian tissue through folliculogenesis and regenerate the endometrium to improve fertility outcomes [15]. When applied to surgical wounds, early studies have suggested that PRP improves wound healing, significantly improving erythema, ecchymosis and oedema, in addition to reducing pain as well as keloid and hypertrophic scar formation [15]. Owing to the minimally invasive method of application and use of autologous solutions, PRP reduces the risk of infection, autoimmune reaction and serious complications when compared with surgical management alone [15].

A recent systematic review [18] assessed the efficacy of CO_2_ laser therapy for GSM symptoms in postmenopausal women, noting safety and efficacy. However, the quality of the included studies was generally rated as "very low" or "low" [18]. Although a recent randomised controlled trial found that fractional CO_2_ laser treatment did not significantly improve vaginal symptoms, it showed that CO_2_ laser does not exhibit significant adverse effects [19]. Mechanistically, CO_2_ laser therapy may rehydrate the tissue and stimulate collagen synthesis [20, 21]. By creating microchannels in the tissue through CO_2_ laser, the absorption of PRP may be enhanced without the need for multiple injection sites, allowing the PRP to penetrate deeper into the tissue, where it can exert its therapeutic effects. When these modalities are combined, it is postulated that their effect is synergistic [22]. It has been proposed that the anabolic effects of PRP, combined with microtrauma and heat from CO_2_ laser, may improve neovascularisation, cell regeneration, elasticity and collagen synthesis [22].

We aim to investigate the synergistic effects of PRP and CO_2_ laser or CO_2_ laser alone in reducing surgery for mesh complications. Additionally, we evaluate the effects of these treatments on bowel, bladder and vaginal function.

## Materials and Methods

**Fig. 1:**
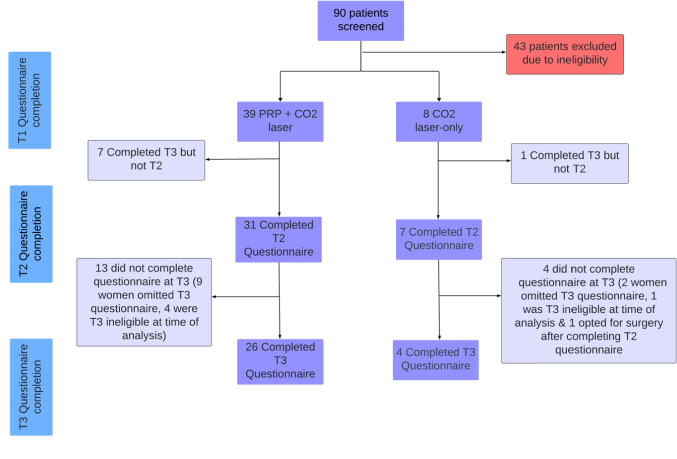
Enrolment flowchart of the study participants

This prospective observational study assesses the efficacy of combined PRP and CO_2_ laser for treating symptomatic vaginal-mesh complications. Ethics approval was obtained (application ID: 2016-04-293-PRE-7). Written consent was obtained from all participants, and an enrolment flowchart is shown in Fig. 1. Participants were recruited consecutively from a single private gynaecology practice receiving referrals for mesh-related complications between 2013 and 2024. Women were deemed eligible to participate if they had implanted urogynaecological mesh and reported symptoms from vaginal mesh such as vaginal bleeding, discharge, pelvic pain, dyspareunia and/or vaginal mesh exposure < 2 cm. Excluded from the study were women who had vaginal mesh complications such as vaginal mesh exposure ≥ 2 cm, visceral mesh erosion, active cancer, a genital-tract fistula, contraindications to PRP, or were pregnant. PRP was contraindicated in individuals with conditions such as thrombocytopenia, severe anaemia, unmanaged diabetes or who were using anticoagulation therapies such as warfarin. Participants were not charged for therapies. The criterion mesh exposure of < 2 cm was chosen because of the extensive clinical experience of the primary gynaecological surgeon with complicated mesh-healing outcomes based on the literature.

The mesh types were primarily polypropylene, AMS mesh or J&J mesh, with symptom duration of at least 3–6 months. The primary outcome of the study was to determine the number of women who did not require surgical intervention for the aforementioned mesh complications following treatment with PRP and CO_2_ laser.

The standard approach to vaginal-mesh exposure includes topical oestrogen, wound care and pelvic floor therapy, with surgery considered if symptoms persisted.

Surgical excision was chosen as the primary outcome because it represents a definitive and objective measure of treatment success. Symptomatic improvement can be highly subjective and influenced by multiple factors, whereas the need for surgical intervention is a clear clinical decision. Surgical excision is typically considered when conservative management fails, making it a more reliable endpoint for evaluating the efficacy of alternative therapies such as PRP and CO_2_ laser treatment.

Ultimately, the decision to proceed with surgery was patient driven, based on the severity of the ongoing discomfort, vaginal discharge or other persistent symptoms that impacted their quality of life. Patients who felt that their symptoms were significant enough to warrant surgical intervention were offered the procedure, ensuring a shared decision-making process between clinician expertise and patient preference.

Secondary outcomes included results from the Australian Pelvic Floor Questionnaire (APFQ), the Pelvic Organ Prolapse Quantification (POPQ) staging system and the severity of vaginal atrophy. Questionnaires were collected at baseline (T1), 3–6 months (T2) and > 9 months (T3) for the PRP and CO_2_ laser combined cohort as well as a second cohort consisting of CO_2_ laser-only participants. The CO_2_ laser-only group either declined PRP or PRP was contraindicated.

The APFQ has established validity in the Australian population assessing pelvic-floor function [23]. This questionnaire consists of four domains, namely bladder, bowel, prolapse and sexual function. Each question is in a Likert-scale format ranging from 0 to 3, where higher scores generally represent greater symptom severity. The APFQ also allows for other symptoms to be qualitatively described within each domain. Participants completed the APFQ at baseline (T1), and at least once post-treatment (a minimum of 3 months after starting treatment).

Mesh-exposure measurements were assessed vaginally in millimetres, at baseline (T1), 3–6 months (T2) and > 9 months (T3). The severity of atrophic vaginitis was clinically determined on standard examination at baseline (T1), 3–6 months (T2) and > 9 months (T3) using a four-point Likert scale (0 = not atrophic, 1 = mild, 2 = moderate, 3 = severe). The POP stage was assessed according to the POPQ system at baseline (T1), 3–6 months (T2) and > 9 months (T3).

### PRP and CO_2_ Laser Technique

Microablative, fractional CO_2_ laser therapy (MonaLisa Touch, SmartXide2 V2LR, DEKA, Italy) was administered vaginally utilising a single mirror probe to avoid discomfort. A single mirror probe directs the laser beam using one reflective mirror, allowing for precise, focused application of energy on a smaller area and more importantly avoiding the laser beam coming into direct contact with the mesh material. This setup typically offers greater control over specific treatment zones. Fractional CO_2_ laser is performed first, creating microchannels, allowing a theoretically greater absorption of non-injected PRP (i.e. poured) and reducing needle injection sites for injected PRP, therefore potentially reducing patient pain and discomfort [24]. An additional advantage of performing fractional CO_2_ laser, prior to the application of PRP therapy, is the potential therapeutic effect of CO_2_ laser in promoting tissue healing at the mesh-exposure site.

Each patient’s whole blood (10 ml) was centrifuged on site for the preparation of PRP. RegenPRP® (Switzerland) tubes were used; the PRP was poured into the vaginal canal immediately after fractional CO_2_ laser treatment, then injected around the mesh (in the case of exposure) and vaginal opening using a 27-gauge needle [16]. Patients underwent three consecutive treatments of either PRP and CO_2_ laser or CO_2_ laser alone at 4– to 6-week intervals. Subsequent treatment of PRP and CO_2_ laser or CO_2_ laser alone was scheduled yearly, as per practice standard, for maintenance and/or symptom management. No prophylactic antibiotics were given, and patients were advised to avoid intercourse and tampon usage for 5 days post-treatment.

### Statistical Analyses

The patient characteristics were analysed using descriptive statistics to provide a comprehensive overview of the study population. The Wilcoxon signed-rank test was used to assess the interquartile (IQR) changes in symptom severity of the APFQ and POPQ. A general linear model with multivariate and repeated measures components was utilised to analyse the differences in outcomes across the three time points (T1, T2 and T3). The multivariate approach was chosen to account for potential correlations among the outcome variables. The repeated measures aspect allowed for the examination of within-subject changes over time. The level of significance was set as a *p* value of < 0.05. All analyses were performed using IBM SPSS Statistics version 29.01.0 (Armonk, NY). Patients and evaluator were not blinded to the treatments and assessments.

## Results

A total of 90 patients with mesh-related complications referred for surgical excision of mesh were identified (Fig. 1). Forty-seven patients met the inclusion criteria, 39 patients underwent PRP and CO_2_ laser combined and 8 patients received treatment with fractional CO_2_ laser alone.

A total of 40 out of 47 patients (85.0 %) had previously used vaginal oestrogen and were non-responsive. There were no PRP and CO_2_ laser equipment failures. There were no cases of post-treatment vaginitis or viscous perforation. Further, there was no reported worsening of symptoms post-treatment (i.e. increase in mesh-exposure size, worse pain, vaginal discharge and/or bleeding). The average follow-up was 12 months.

Demographic and clinical characteristics of the PRP and CO_2_ laser-combined group, and the CO_2_ laser-only group is provided in Table 1. The average age of the PRP and CO_2_ laser-combined group was 62.74 ± 9.15 years and the average BMI was 28.22 ± 6.29 kg/m^2^ (Table 1). Owing to the sample size (*N* = 8) within the fractional CO_2_ laser-only group, statistical-significance analysis across groups was deemed inapplicable. Table 2 demonstrates descriptive analysis of APFQ categories for the CO_2_ laser-only treatment group.

**Table 1:** Demographic and clinical characteristics of the patient cohort

Patient demographic and clinical variables	PRP and CO_2_ laser group (*N* = 39)	CO_2_ laser only group (*N* = 8)
Age (years), mean ± SD	62.74 ± 9.15	70.63 ± 12.02
BMI (kg/m^2^), mean ± SD	28.22 ± 6.29	26.84 ± 6.71
Parity, median (minimum–maximum)	2 (0–4)	2.5 (0–5)
Parity (≥ 1), *n* (%)	33 (84.6)	5 (62.5)
Vaginal birth (≥ 1), *n* (%)	31 (79.5)	4 (50.0)
Vaginal birth, median (minimum–maximum)	2 (0–4)	2 (0–5)
Oestrogen cream use, *n* (%)	32 (82.1)	8 (100.0)

*BMI* body mass index, *SD* standard deviation, *PRP* platelet-rich plasma

**Table 2:** Statistical analysis of Australian Pelvic Floor Questionnaire (APFQ) categories of CO_2_ laser-only treatment group (*N* = 8)

Variable	Descriptive statistics
Bladder score, mean ± SD	
T1 (*n* = 8)	10.00 ± 5.85
T2 (*n* = 7)	7.38 ± 6.45
T3 (*n* = 4)	9.80 ± 8.34
Bowel score, mean ± SD	
T1 (*n* = 8)	12.25 ± 3.53
T2 (*n* = 7)	10.00 ± 4.95
T3 (*n* = 4)	7.00 ± 8.71
Prolapse score, mean ± SD	
T1 (*n* = 8)	4.88 ± 4.97
T2 (*n* = 7)	2.75 ± 3.77
T3 (*n* = 4)	4.25 ± 6.65
Sexual score, mean ± SD	
T1 (*n* = 8)	3.25 ± 5.12
T2 (*n* = 5)	1.13 ± 1.55
T3 (*n* = 2)	0.75 ± 1.50
POPQ, mean ± SD	
T1	1.33 ± 1.52
T2	1.00 ± 1.00
T3 (*n* = 2)	1.50 ± 2.12
Atrophic vaginitis severity, mean ± SD	
T1	2.50 ± 0.71
T2	0.67 ± 0.57
T3 (*n* = 2)	0.50 ± 0.71

The primary outcome of reducing surgical intervention for bothersome mesh complications such as dyspareunia, vaginal discharge and mesh exposure < 2 cm was defined by clinical speculum and digital examination. The majority (33 out of 39, 84.6%) of the PRP and CO_2_ laser group and 7 out of 8 of the CO_2_ laser-only group (87.5%) did not require surgical intervention after completing three treatments.

**Fig. 2:**
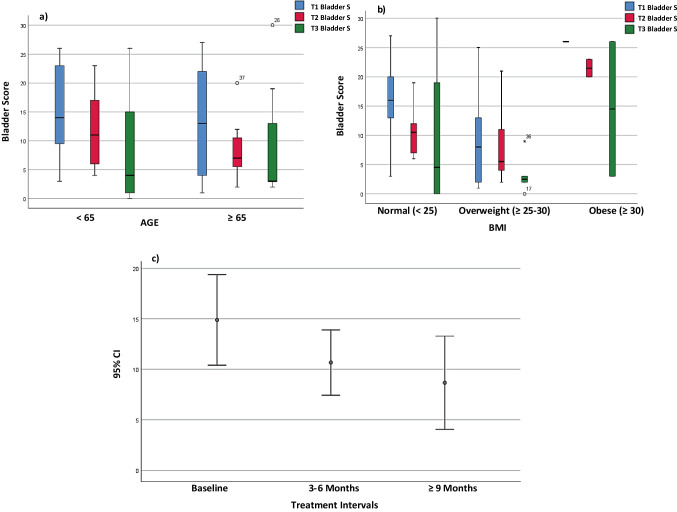
Distribution of overall Australian Pelvic Floor Questionnaire bladder scores at Baseline (T1), 3–6 months (T2) and > 9 months (T3) by **a** age (*p* = 0.42), **b** body mass index (BMI) (*p* = 0.23) and **c** treatment interval (*p* < 0.001) in the platelet-rich plasma and CO_2_ laser group

The APFQ was completed by the entire (100.0%) PRP and CO_2_ laser combined group of women at T1, 32 out of 39 (82.0%) of the women at T2 and 26 out of 39 (66.7%) of the cohort at T3. The distribution of overall APFQ bladder function score by age and BMI is shown in (a and b). Figure 2c compares the distribution of overall bladder function score between baseline (T1), 3–6 months (T2) and ≥ 9 months (T3) (*df* = 1, *F* value = 77.28, *p* < 0.001). After controlling for age and BMI, the differences observed for overall bladder function scores at each time point significantly improved (*df* = 2, *F* value = 7.57, *p* = 0.005). There were significant median changes in Q4, urinary urgency (T2 vs T1 *p* = 0.01); Q5, urge urinary leakage (T2 vs T1 *p* = 0.03); Q6, stress urinary leakage (T2 vs T1 *p* = 0.01); Q11, fluid intake limitation to reduce urinary leakage (T2 vs T1 *p* = 0.006; T3 vs T1 *p* = 0.01); and Q15, bladder-function impact on quality of life (T2 vs T1 *p* = 0.02).

**Fig. 3:**
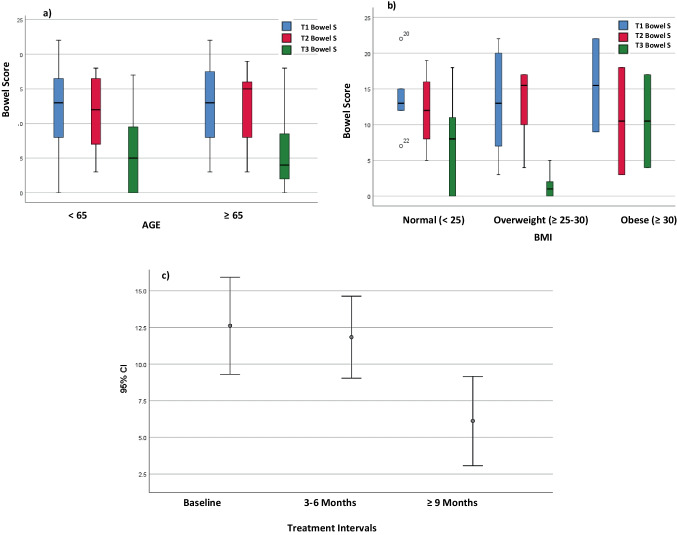
Distribution of overall Australian Pelvic Floor Questionnaire bowel scores at Baseline (T1), 3–6 months (T2) and > 9 months (T3) by **a** age (*p* = 0.75), **b** body mass index (BMI; *p* = 0.40) and **c** treatment interval (*p* < 0.001) in the platelet-rich plasma and CO_2_ laser group

The distribution of overall APFQ bowel function scores by age and BMI is shown in Fig. 3a and b. In Fig. 3c overall bowel scores improved after treatment (*df* = 1, *F* value = 64.07, *p* < 0.001). After controlling for BMI and age, the overall bowel score differences observed at different time points remained significant (*df* = 2, *F* value = 11.39,* p* = 0.001). Statistically significant median changes in APFQ bowel symptom severity included Q18, strain to empty bowel (T3 vs T1 *p* = 0.03); Q20, constipation (T2 vs T1 *p* = 0.01; T3 vs T1 *p* = 0.02); Q25, incomplete emptying (T3 vs T1 *p* = 0.01); Q26, digital pressure assisting rectal emptying (T2 vs T1 *p* = 0.02; T3 vs T1 *p* = 0.02); and Q27, impact of bowel symptoms on daily life (T3 vs T1 *p* = 0.01).

**Fig. 4:**
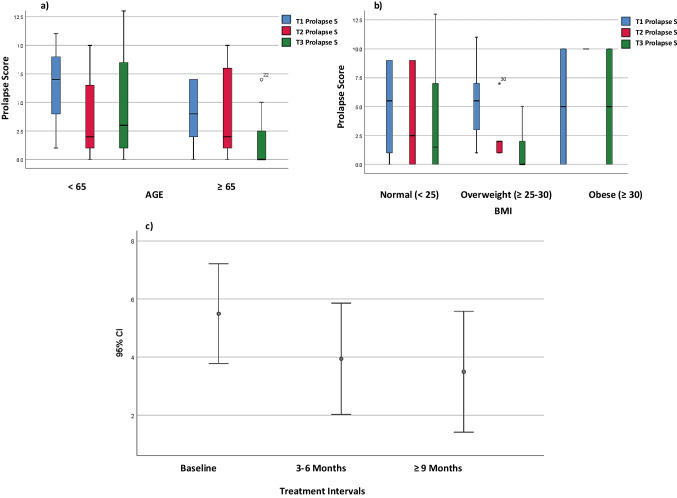
Distribution of overall Australian Pelvic Floor Questionnaire prolapse scores at Baseline (T1), 3–6 months (T2) and > 9 months (T3) by **a** age (*p* = 0.39), **b** body mass index (BMI; *p* = 0.10) and **c** treatment interval (*p* = 0.002) in the platelet-rich plasma and CO_2_ laser group

The distribution of overall APFQ prolapse scores by age and BMI is shown in Fig. 4a and b. In Fig. 4c prolapse symptom scores significantly decreased post-treatment (*df* = 1, *F* value = 20.45, *p* = 0.002). Significant difference between time points was not visible after controlling for BMI and age (*df* = 2, *F* value = 2.05, *p* = 0.16). Statistically significant median changes in APFQ prolapse symptoms improved in Q28, prolapse sensation (T2 vs T1 *p* = 0.01; T3 vs T1 *p* = 0.005); Q29, sensation of vaginal heaviness (T3 vs T1 *p* = 0.009); and Q32, impact of prolapse symptoms on daily life (T2 vs T1 *p* = 0.03; T3 vs T1 *p* = 0.03). POPQ staging decreased between T2 vs T1 (*p* = 0.003) and T3 vs T1 (*p* = 0.001).

**Fig. 5:**
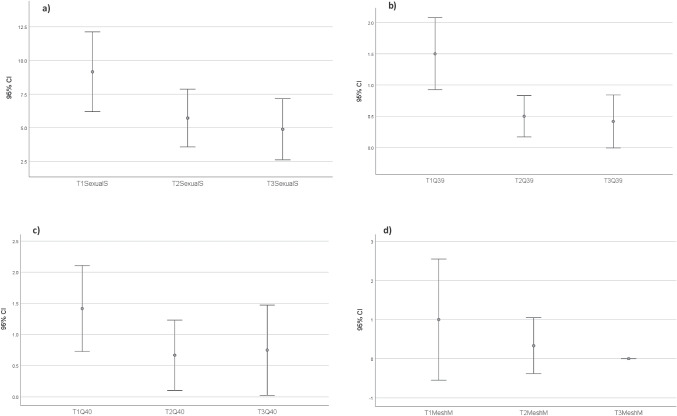
Overall Australian Pelvic Floor Questionnaire scores in the platelet-rich plasma and CO_2_ laser group at Baseline (T1), 3–6 months (T2) and > 9 months (T3) for **a** Sexual function (*p* < 0.001), **b** dyspareunia (*p* = 0.02), **c** vaginal pain location (*p* = 0.02) and **d** mesh measurements (T3 *p* = 0.02 but T2 *p* = 0.2)

As shown in Fig. 5a, overall APFQ sexual function scores improved after treatment (*df* = 1, *F* value = 45.88, *p* < 0.001). BMI and age did not have an effect on this significant difference between three time points (*df* = 2, *F* value = 7.53, *p* = 0.004). Significant differences in dyspareunia (Fig. 5b), were observed across various time points (*df* = 1, *F* value = 20.71, *p* = 0.02). The location of vaginal pain (Fig. 5c, significantly improved over time (*df* = 1, *F* value = 17.00, *p* = 0.02). In Fig. 5d transvaginal mesh exposure measurement showed a significant improvement when comparing T3 with T1 (*p* = 0.02). However, T2 did not exhibit a significant change compared with T1 (*p* = 0.2). Statistically significant median changes in APFQ sexual-function scores were shown in Q36, vaginal sensation during intercourse (T2 vs T1 *p* = 0.007; T3 v T1 *p* = 0.02); Q37, vaginal laxity (T3 vs T1 *p* = 0.04); Q39, dyspareunia (T2 vs T1 *p* = 0.01; T3 vs T1 *p* = 0.004); Q40, pain location during vaginal intercourse (T2 vs T1 *p* = 0.01); and Q42, impact of sexual issues on daily life (T2 vs T1 *p* = 0.004; T3 v T1 *p* = 0.009).

## Discussion

The findings of this study support that after completing three treatments of PRP and CO_2_ laser or CO_2_ laser-alone, 40 out of 47 of the cohort (85.0%) did not require surgery to address symptoms related to mesh complications. The study also found improvements in mesh-exposure measurements at T3 vs T1, as well as APFQ scores for urinary, bowel, prolapse and sexual function over the study period. Additionally, there were improvements in dyspareunia and vaginal pain location. Statistical analysis revealed significant improvements in three out of four domains after adjusting for age and BMI. However, prolapse scores, after adjusting for these factors, were no longer statistically significant. This suggests that higher BMI and advanced age might play a crucial role in the development and persistence of prolapse-related issues [23].

There is a scarcity of data on the treatment options for mesh complications [25]. Topical vaginal oestrogen and systemic antibiotics are considered first-line interventions but the failure rate is high [26]. A minimally invasive approach to mesh explantation is recommended for patients with severe symptoms. However, explantation fails to relieve symptoms in 15.0% of women with mesh complications [27]. Although the current study provides encouraging evidence for a non-excisional approach to vaginal mesh complications, more research is needed to determine its effectiveness compared with selective excision and total explantation.

Platelet-rich plasma therapy, either as a standalone approach or in combination with fractional CO_2_ laser, has been utilised for the treatment of pelvic-floor dysfunction in women without mesh implants. Several systematic reviews and trials have investigated the effectiveness of these modalities in addressing GSM, lichen sclerosis and urinary incontinence [15–18, 21, 28, 29]. These studies have reported significant improvements in bladder and/or sexual function, aligning with the observations made in the present study [30]. Although studies have not shown significant improvement for postmenopausal vaginal symptoms, they have found no severe adverse outcome from fractional CO_2_ laser treatment [20]. In light of these findings, it is important to underscore the supportive role of fractional CO_2_ laser therapy when applied to trauma sites, working in conjunction with PRP treatment in a painless manner utilising the microchannels created by fractional CO_2_ laser. This combination approach holds potential for enhancing the therapeutic effects of PRP therapy in the management of mesh exposure.

The study included two treatment groups: PRP + CO_2_ laser and CO_2_ laser alone, with allocation based on patient preference and contraindications to PRP. Key factors influencing group assignment included religious beliefs (e.g., Jehovah’s Witnesses avoiding PRP), needle phobia and a preference for CO_2_ laser based on perceived evidence. This patient-centred approach enhances clinical relevance and allows for a comparative analysis of PRP augmentation versus laser alone in managing mesh exposures.

Limitations of this study are the small sample size, the lack of a control group or comparator and a 34.0% loss to follow-up at > 9 months. Also, the needling method used to inject PRP intravaginally may itself stimulate vaginal healing. Therefore, a double-blinded randomised control trial (RCT) could determine the potential benefit of needling, if any, and further assess the efficacy of these modalities in a larger group of patients with complications from urogynaecological mesh.

The advantages of this treatment are that it is office-based, has a short recovery time and a low rate of complications. The results of this study suggest that regenerative medical therapies might improve the health of vaginal mucosa and reduce the effects of complications related to transvaginal mesh.

## Conclusions

The combined use of fractional PRP and CO_2_ laser showed a symptom improvement and reduced need for surgical excision as well as improvement in pelvic-floor dysfunction including bladder, bowel, sexual function and prolapse symptoms. A significant majority of the cohort, 40 out of 47 participants (85.0%), did not require subsequent surgical intervention after three treatments of either PRP and CO_2_ treatment or CO_2_ laser-only. These modalities may act synergistically by inducing inflammation and promoting wound healing. Further studies such as RCTs with larger cohorts are required to clarify their therapeutic role.

## Data Availability

The datasets generated during and/or analysed during the current study are available from the corresponding author on request.
